# Draft Genome Sequence of *Acinetobacter* sp. HR7, Isolated from Hanwoo, Korean Native Cattle

**DOI:** 10.1128/genomeA.01358-14

**Published:** 2015-01-08

**Authors:** Dong-Ho Chang, Moon-Soo Rhee, Haeyoung Jeong, Seil Kim, Byoung-Chan Kim

**Affiliations:** a Korean Collection for Type Cultures, Biological Resource Center, Korea Research Institute of Bioscience and Biotechnology (KRIBB), Yuseong-Gu, Daejeon, Republic of Korea; b Korean Bioinformation Center, KRIBB, Yuseong-Gu, Daejeon, Republic of Korea; c Division of Metrology for Quality of Life, Center for Bioanalysis, Korea Research Institute of Standards and Science (KRISS), Yuseong-Gu, Daejeon, Republic of Korea

## Abstract

*Acinetobacter* species have been reported as opportunistic pathogens. Here, we report the draft genome sequence of *Acinetobacter* sp. HR7 isolated from the rumen of cannulated Korean native cattle (Hanwoo; *Bos taurus coreanae*).

## GENOME ANNOUNCEMENT

The genus *Acinetobacter* comprises aerobic, nonfermenting, nonmotile, and Gram-negative coccobacilli. It was first described by Brisou and Prévot in 1954 and currently consists of 33 distinct species with validly published names (http://www.bacterio.cict.fr/a/acinetobacter.html). *Acinetobacter* species have been isolated in nature, such as in soil, water, dry environments, and on hospital equipment (1). Certain *Acinetobacter* species have emerged as pathogens that have been found in intensive care units (ICUs) (2). In the course of investigating the microbial diversity of the rumen of Korean native cattle and isolating culturable microbes, a novel *Acinetobacter* sp. was isolated from fluid in the rumen. Recently, *Acinetobacter* sp. was reported to be found in the rumens of dairy cattle through a pyrosequencing process (3, 4). However, no species have been isolated and/or identified in cattle rumen thus far. In this article, we announce the draft genome sequence of *Acinetobacter* sp. HR7. It is the first draft genome sequence of the genus *Acinetobacter* sp. to originate from a bovine rumen.

The HR7 genome was sequenced using an Illumina HiSeq 2000 system at the Genome Research Center of the Korea Research Institute of Bioscience and Bioengineering (KRIBB). A total of 12,307,475 paired-end reads (396.8-fold coverage) were obtained from the HiSeq 100-bp paired-end library and were preprocessed and *de-novo*-assembled using the CLC Genomics Workbench (CLC bio), version 7.5. The genome sequence was assembled into 221 scaffolds (227 contigs). The sizes of the largest and the *N*_50_ contigs were 113,099 and 27,989 bp, respectively. The open reading frames (ORFs) of the assembled genome were predicted and annotated using the Integrated Microbial Genomes—Expert Review (IMG-ER) (5), NCBI Clusters of Orthologous Groups (COG) (6), Pfam (7), and EzTaxon-e (8) databases, and the rRNA genes and tRNA genes were identified with the RNAmmer 1.2 (9) and tRNAscan-SE 1.23 (10) tools, respectively. The draft genome of *Acinetobacter* sp. HR7 was 3,132,425 bp. The G+C content was 43.5%, and ORF were 2,938. The numbers of tRNA and rRNA, and the protein coding genes with functions were 75, 3 and 1,682, respectively.

Based on the annotation results, 5 genes were found to be related to multidrug resistance and one gene is coded for beta-lactamase. One mechanism of resistance to beta-lactams of *A. baumannii* is considered to be the expression of oxacillinases (OXA) for hydrolyzing carbapenem (11) and a gene coding OXA-like protein was also found in the HR7 genome. These findings indicate that the isolate is resistant to multiple types of antibiotics. In addition, a putative siderophore biosynthesis operon and three genes for TonB-like proteins related to ferric siderophore transport systems were found in the genome, indicating that the isolate could be a pathogen because the expression of siderophores is thought to be one of the pathogenic mechanisms of *A. baumannii* infection (12). The partial sequence of the16S rRNA gene of the isolate showed 98.1% similarity to that of *A. schindleri*. The potential pathogenicity of the novel *Acinetobacter* sp. HR7 from the bovine rumen requires further study.

### Nucleotide sequence accession numbers.

The draft genome sequence of *Acinetobacter* sp. HR7 is available in DDBJ/EMBL/GenBank under the accession no. JPQO00000000. The partial sequence of the 16S rRNA gene of *Acinetobacter* sp. HR7 has been deposited into the GenBank database under the accession no. KJ670315.1.
